# A Critical Appraisal of Quantitative Studies of Protein Degradation in the Framework of Cellular Proteostasis

**DOI:** 10.1155/2012/823597

**Published:** 2012-10-21

**Authors:** Beatriz Alvarez-Castelao, Carmen Ruiz-Rivas, José G. Castaño

**Affiliations:** ^1^Departamento de Bioquímica, Instituto de Investigaciones Biomédicas “Alberto Sols” (UAM-CSIC), Centro de Investigación Biomédica en Red sobre Enfermedades Neurodegenerativas (CIBERNED), Facultad de Medicina, Universidad Autónoma de Madrid, 28029 Madrid, Spain; ^2^Departamento de Matemáticas, Facultad de Ciencias, Universidad Autónoma de Madrid, Cantoblanco, 28049 Madrid, Spain

## Abstract

Protein homeostasis, proteostasis, is essential to understand cell function. Protein degradation is a crucial component of the proteostatic mechanisms of the cell. Experiments on protein degradation are nowadays present in many investigations in the field of molecular and cell biology. In the present paper, we focus on the different experimental approaches to study protein degradation and present a critical appraisal of the results derived from steady-state and kinetic experiments using detection of unlabelled and labelled protein methodologies with a proteostatic perspective. This perspective allows pinpointing the limitations in interpretation of results and the need of further experiments and/or controls to establish “definitive evidence” for the role of protein degradation in the proteostasis of a given protein or the entire proteome. We also provide a spreadsheet for simple calculations of mRNA and protein decays for mimicking different experimental conditions and a checklist for the analysis of experiments dealing with protein degradation studies that may be useful for researchers interested in the area of protein turnover.

## 1. Introduction: Cellular Proteostasis

**Figure 1:**
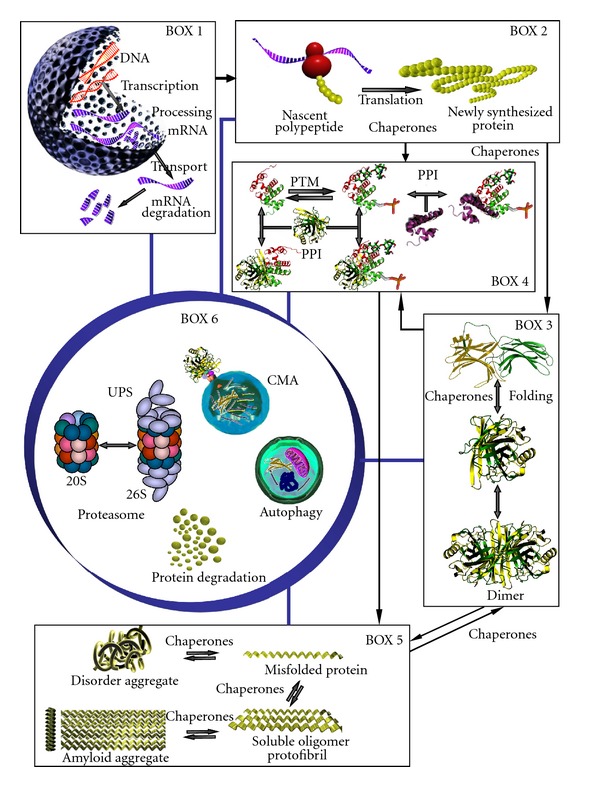
Schematic diagram of cell proteostasis. The boxes illustrate the different cellular process involved in protein homeostasis. BOX 1, nucleus, where gene transcription and pre-mRNA processing produce the mature mRNA that will be transported to the cytoplasm where it could be degraded (mRNA decay can also take place in the nucleus). The mRNA is engaged, mainly in the cytoplasm, to translation by the ribosomal machinery producing a nascent polypeptide that grows to a newly synthesized protein (BOX 2). The folding of the newly synthesized proteins, helped by chaperones, results in the “so-called” native protein structure either monomeric or oligomeric (BOX 3, only a dimer is shown for simplicity). Both the newly synthesized proteins and the mature mono or oligomeric forms of proteins are subjected to post-translational modification (PTM) and specific and unspecific protein-protein interactions (PPIs) that are illustrated in BOX 4. Proteins due to changes in its native conformation produced by different physical and/or chemical perturbations of the cell, or by mutations, could get misfolded or misprocessed and misfolded. The misfolded proteins, perhaps under the influence of PPIs and PTMs, will produce protein aggregates or soluble oligomeric protofibrils that eventually may form amyloid fibers (BOX 5). The degradation of proteins (BOX 6) is mainly due to the ubiquitin-proteasome System (UPS, nucleus and cytoplasm, 20S, and 26S proteasome) and the autophagic pathways (cytoplasmic): mainly chaperone-mediated authopagy (CMA) and macroautophagy (autophagy). Other proteases also participate in protein degradation (calpains, caspases, etc.), not shown. Blue lines connect all the boxes to the central circular box of protein degradation (BOX 6) indicating that proteolysis can regulate any of the process and vice versa. Black arrows connecting boxes indicate the “flow” of the products depicted in each BOX.

The living cell requires a homeostatic control of energy, use, and production to accomplish the different cell functions. Proteins are the main producers, users and transformers of energy. The set of proteins that are present in a cell at a given time is what we call the cell proteome. The cellular proteome has to take care of itself and its behaviour determines cell function. Accordingly, the proteome has its own homeostasis that is necessarily coupled, at least, to energy homeostasis. Protein homeostasis, proteostasis, is critical for the adaptation of cell function to a fluctuating internal and external milieu. Those adaptative responses, like regular exercise for us, keep the proteome in good “shape.” The proteostatic mechanisms of a cell involve a complex network of pathways that includes protein synthesis, folding, posttranslational modifications (PTMs), protein-protein interactions (PPIs), subcellular localization, and degradation. Protein expression levels in eukaryotes are determined by several processes, beginning with nuclear gene expression. Nuclear gene transcription, pre-mRNA processing, mRNA nuclear transport, and degradation (Figure 1, BOX 1) are the initial steps determining the available pool of cell mRNAs that can be translated, the translatome, the total mRNAs that are in ribosome complexes undergoing translation (Figure 1, BOX 2). The life of a protein begins as a nascent polypeptide by translation of its mRNA (Figure 1, BOX 2). The “survival” or “demise” of the nascent polypeptides and the newly synthesized proteins is under control. Many cellular proteins can be degraded at this early stage of its biogenesis, including those that are defective that constitutes the so-called defective ribosomal products (DRiPs) and that could potentially account for up to 30% of the polypeptides synthesized by a mammalian cell [1]. In this early stage, correct folding of the newly synthesized proteins to its functional tertiary and quaternary structures (Figure 1 BOX 3) is assisted by dedicated chaperones that also play an important role in reverting misfolding [2]. Specific PTMs and PPIs of the nascent, newly synthesized or the mature native proteins are due to their living circumstances in a general crowded environment controlled by thermal motion and diffusion rates (Figure 1, BOX 4) with an estimated protein concentration close to 30 mM [3]. Due to physical and/or chemical modification, native proteins may get misfolded given rise to the formation of aggregates or protofibrils which eventually organized into amyloid fibers (BOX 5). The ubiquitin proteasome and autophagic pathways (Figure 1, BOX 6) are the main pathways of general protein degradation. PTMs and PPIs together with subcellular localization determine protein degradation by those pathways. The subcellular localization of proteins certainly contributes to their proteostasis: protein trafficking, folding, and regulation of degradation of the specific translatome for mitochondria (chloroplasts), the endoplasmic reticulum, and the secretary pathway; the peroxisomes and the cell nucleus have mechanistic differences from the cytoplasmic protein proteostasis [4–8]. In addition, the localization of mRNAs and the translational machinery in the cell are also highly relevant to proteostasis. A special case is neurons that have specialized compartmentalization, dendrites, and axons. Transport of some mRNAs and the translational machinery to those distant places from the neuronal cell soma and the retrograde transport to the soma are crucial step to maintain proteostasis at dendrite and axons, but they may also exist proteostatic mechanisms at those locations likely to be both quantitatively and qualitatively different from soma proteostasis [9, 10]. Finally, cellular proteostasis may be controlled by signalling pathways in a cell-nonautonomous manner that remain to be identified [11].

The energetic cost of the production and maintenance of a healthy proteome is critical to determine life sustainability and is probably one of the reasons why protein and energy homeostasis have coevolved to allow the appearance of complexity. Accordingly, their regulatory mechanisms have important crosstalks and are well conserved during evolution [12]. Energy and/or protein homeostasis failure leads to cell dysfunction and eventually to cell death, as may occur during aging, neurodegenerative diseases [13], and possibly in other chronic human diseases; diabetes, cancer, renal and pulmonary fibrosis, and so forth. Certain proteins, like toxic metabolites, can become proteotoxic and the cell has also mechanisms to cope with those toxic proteins (Figure 1, BOX 5), in particular for misfolded or aggregated proteins [14]. The global connection of energy and protein homeostasis is also supported by recent experiments showing that significant increase of life span results from reduction of insulin/IGF signaling or by loss of VHL/HIF1, that also improves function of transgenic models of proteotoxic diseases as reported for *C. elegans* [15, 16] and mice [17].

Studies on protein turn-over are used by many researchers in many different areas of cellular and molecular biology, but sometimes the interpretations of the results are leading to incorrect conclusions. This paper deals with the experimental approaches used to study protein degradation in a quantitative basis and how a critical appraisal of the interpretations of the results in the framework of proteostasis would certainly help to arrive to appropriate conclusions, knowing what we can reasonably conclude from each experimental setup.

## 2. Steady-State versus Kinetic Studies

By definition, the steady-state level of each protein of a cell proteome is attained when its rate of synthesis and its rate of degradation are equal, invariant respect to time. Also by definition, except in the quiescent state, cells will grow and divide generating two daughter cells; as a consequence, the entire proteome is diluted by the increase in cell volume due to cell division cycle. Each protein of the proteome will have its own steady-state levels, and changes in the steady-levels are the result of changes in the rate of protein synthesis and/or the rate of protein degradation.

The rate of synthesis of the entire proteome of a cell is controlled by the relative abundance of the different mRNAs of the transcriptome, whose different steady-state levels are also the result of mRNA synthesis and degradation, and by their translational rate. The actual amount of a protein being synthesized is the product of the copy number of mRNA molecules and the number of protein molecules synthesized by unit time per mRNA molecule. The rate of degradation of mRNAs or proteins is generally assumed to follow an exponential decay, similar to the familiar radioactive decay of an isotope. Accordingly, the changes over time of the amount of a cell protein can be expressed with the following differential equation, taken from Belle et al. [18],

(1) $$\begin{array}{c} P\left(t\right)=\frac{dP}{dt}=M\left(t\right)*R-P\left(t\right)*\left(D+V\right), \end{array}$$

where *P* is the protein concentration, *M* is the copy number of the mRNA, *R* is the rate of translation per mRNA molecule, *D* is the protein degradation rate constant, and *V* is the growth rate (volume increase factor per unit time). At steady state, the rate of protein synthesis and degradation are equal, and as a consequence, *dP*/*dt* = 0, no change in the amount of protein over time. The steady-state levels of a protein can be defined by the following equation, also taken from Belle et al. [18],

(2) $$\begin{array}{c} \left(P0\right)=\frac{M\left(0\right)*R}{D+V}. \end{array}$$

We provide an on-line spreadsheet that allows for easy graphical visualization of the rate of protein degradation after the introduction of different values for the terms of the differential equation: number of RNA molecules, rate of mRNA translation, mRNA half-life, protein half-life, and cell doubling times.

Nowadays, the heavy/light isotope pulse-labelling approach, stable isotope labeling by amino acids in cell culture (SILAC), followed by mass spectrometry- (MS-) based quantitative proteomics, is the method of choice to measure protein turn-over rates under steady-state conditions [19, 20] and in a proteomic scale. The SILAC/MS methodologies when possible, and others like the isobaric tag for relative and absolute quantification (iTRAQ), are presently used to measure degradation rates (half-lives) of proteins. These methodologies can also be used to compare relative protein abundance in cells that have been subjected to a stimulus, moving from one to another steady state and even for single cell analysis [21]. New technical developments are being designed that may allow the inclusion of the study of PTMs effects on protein turn-over changes in response to a cell stimulus or stress [22]. Nevertheless, more “traditional” methods to measure protein turn-over are still widely used. There are two basic experimental designs: measuring the amount of a certain protein before and very long after a cell perturbation and measuring the changes in protein concentrations by kinetic, time-course experiments. In the first approach, enough time is provided after cell perturbation to ensure that the cell moves from the initial to a new steady state. In this steady-state situation, the observed changes in protein levels can be attributed to either changes in protein synthesis or degradation, or to both, changes in synthesis and degradation. In the kinetic approach, time-course experiments, the contribution of each of the branches (synthesis and degradation) to the new steady state after cell perturbation is studied. The analysis of these approaches will be the focus of the following sections of this paper.

## 3. Pharmacological and Genetic Interference Studies of Protein Degradation

The most common experimental setup to study protein degradation is to measure changes in protein levels mainly by protein immunoblot of total cell extracts. The pharmacological interference to study protein degradation is performed by treatment of cells with protein synthesis and/or degradation inhibitors. Genetic interference to study protein degradation targets either transcription or mRNA function. Transcriptional downregulation of mRNA expression is achieved by the use of Tet-on or Tet-off transcriptional control of the corresponding gene. The function of mRNA is targeted by specific siRNA or shRNA that results in RNA decay and/or inhibition of protein synthesis of the targeted mRNA.

### 3.1. Pharmacological Interference Studies of Protein Degradation

One of the simplest experiments is to add an inhibitor of protein degradation to the cells, wait overnight or 24 h, and then compare the levels of the protein of interest between the untreated and the treated cells by simple immunoblot of total cell extracts. When the results obtained show an increase in the protein levels, the usual conclusion is that the protein is being degraded by the degradation pathway that is “specifically” blocked by the inhibitor. When there is no change or a decrease in the protein levels, the particular pathway of degradation blocked by the specific inhibitor does not participate in the degradation of the protein. Those conclusions seem appropriate, but can be incorrect. Most of those experiments are performed after prolonged incubation times (as mentioned above), and as a consequence, they are measuring changes in the “steady-state” levels of a protein. The new steady-state level of a protein after treatment with an inhibitor of protein degradation can be reached kinetically by changes in its degradation rate with a constant rate of synthesis, by changes in its rate of synthesis, or by both changes in synthesis and degradation. As a consequence, in those experiments further experiments are needed, before reaching a conclusion. Those controls should include the study of the possible effects of inhibitors of protein degradation on the rate of synthesis of the protein under study. Specifically to measure changes in the transcription (or decay) of the corresponding mRNA that may result in an increase (decrease or no change) in the number of RNA molecules being translated or even the actual rate of mRNA translation in the presence and in the absence of the protein degradation inhibitor.

The aforementioned situation is very clearly exemplified by the broadly used proteasome inhibitors. Those inhibitors are “very specific,” but the ubiquitin-proteasome degradation pathway is also implicated in transcriptional control. The ubiquitin-proteasome pathway participates in the degradation of transcription factors limiting the transcriptional output, and also in recycling of transcriptional complexes on chromatin to facilitate multiple rounds of transcription [23–25] affecting the amounts of mRNA available for translation. As a consequence, if the gene encoding the protein whose degradation is being studied is controlled at the transcriptional or posttranscriptional levels (mRNA processing, nuclear export, mRNA decay, or translation) by the ubiquitin-proteasome pathway, part of the change in steady-state protein levels could be explained by modulation of those upstream mechanisms (Figure 1, affected processes depicted in BOXES 1 and 2). Those upstream processes become especially relevant for proteins with a short half-life (minutes to a few hours). We have shown that transcriptional upregulation of the CMV promoter is the major cause of the increased protein levels of unstable fluorescent reporter proteins after treatment of cells with proteasome inhibitors, and not as much to its decreased rate of degradation by inhibition of the proteasome [26]. The steady-state levels of the unstable fluorescent proteins under basal conditions are very low for a given rate of synthesis and degradation (steady state), the increase in the amounts of the unstable protein by treatment with proteasome inhibitors cannot be accounted by the same rate of protein synthesis as in the basal conditions and simple inhibition of the degradation of the unstable protein. In fact, the increase in protein levels of unstable fluorescent proteins in the presence of proteasome inhibitors can be prevented by cotreatment of the cells with transcriptional inhibitors [26]. The same caveat also applies for proteins with longer half-lives, but in this case the effect observed may be masked due to the higher abundance of those proteins under steady-state conditions that makes more difficult to observe significant changes by immunoblotting.

A similar situation applies to the interpretation of the effect of proteasome inhibitors in time-course experiments. As an example from published results, let us analyze recent papers on the effect of proteasome inhibitors on the ubiquitinome (total ubiquitylated proteins of a cell) protein landscape. Two recent studies [27, 28] state that changes in the abundance of the ubiquitinome are not reflective of overt protein accumulation in response to addition of proteasome inhibitors to the cells. This statement does not mean that there are no clear changes in the abundance of different proteins. In the time-course study of HCT116 cells treated with 1 *μ*M bortezomib reported by Kim et al. [27], the average change in protein accumulation is 1.5-fold after 8 h of treatment (for more than 4500 and less than 5744 protein being analyzed). The corresponding average increase for ubiquitylated modified peptides is 2.8-fold. As the authors already point out that they could not analyze many known low-abundance canonical ubiquitin-proteasome targets, their conclusion should be taken with caution. Further cautions apply to those studies; do not get blinded by high numbers and the use of log2 scales for expressing the results. An average increase of 1.5-fold in the total abundance of the “proteome” after treatment of cells with proteasome inhibitors for 8 h is a tremendous energetic burden for the cell proteostasis, mainly energy expenditure for protein synthesis and folding, and a small fraction of this accumulation could be explained by inhibition of protein degradation (see below). These results clearly show the potent effect of proteasome inhibitors on global regulation of gene expression, but the details are also interesting. For example, the key enzyme controlling glycolysis, phosphofructokinase (PFK), has a half-life of 40–70 h (depending on the isozyme) as recently determined by a SILAC-MS proteomic studies in HeLa and C2C12 cells [29], and is not expected to be very different in HCT116. Accordingly, treatment of cells with 1 *μ*M bortezomib for up to 8 h should not change significantly the total amount of PFK in the cell by inhibition of its degradation. In contrast, Kim et al. [27] report that the abundance of PFK isozymes increase threefold (1.5 on log2 scale used by the authors) after 8 h of incubation of the cells with the proteasome inhibitor. These changes have obvious consequences from the point of view of the regulation of glycolysis and can only be explained by increased protein synthesis, likely due to increased gene transcription, pre-mRNA processing or translation of the corresponding PFK mRNAs. Kim et al. [27] show an increase in HIF1*α* levels by immunoblot analysis, not observed by the SILAC-MS experiments because HIF1*α* is a low abundance protein not detected with the proteomic technologies. Accordingly, the changes in PFK protein levels could be attributed, at least in part, to an increase in gene transcription due to the stabilization of HIF1*α* by proteasome inhibitors, as HIF1*α* is a well-known activator of PFK gene transcription during hypoxia [30]. The effects reported by Udeshi et al. [28] after treatment of the cells for 4 h with 5 *μ*M of a less specific proteasome inhibitor, MG132, are not as extensive in the whole proteome but still are highly significant. The expected effects on protein levels by treatment of cells with proteasome inhibitors are time-dependent as transcription, processing, and translation are needed before a change in protein levels can be observed, and 4 h is enough time to observe those effects translated into protein levels.

The above analysis further strength our conclusion, always consider possible effects on transcription and translation in the interpretation of the results obtained when using inhibitors of protein degradation, as exemplified with proteasome inhibitors. The rational of this consideration seems obvious for experiments with a prolonged time of incubations (steady state) but also applies for time-course experiments that require >2–4 h of treatment of the cells with those inhibitors to observe a significant effect in protein levels. A similar scenario is encountered in experiments where the effects of so-called “toxic” proteins accumulated in neurodegenerative diseases are studied. Those “toxic” proteins have clear effects on gene transcription and posttranscriptional regulation of mRNA, independent of their possible effects on protein degradation [31–33]. In summary, the contribution of changes in the copy number of mRNA molecules and translational rate has always to be taken into account for the correct interpretation of the results of experiments with protein degradation inhibitors or expression of “toxic” proteins under steady-state conditions and in time-course experiments.

The most common kinetic experiments are usually performed by treatment of cells with protein synthesis inhibitors (cycloheximide, emetine, and anisomycin) provided that cell toxicity is controlled by the analysis of cell viability along the time course of the experiment. In this experimental approach, translation is surely inhibited, but also RNA pol I and RNA pol III transcription are inhibited, without affecting general RNA pol II transcription [34], actually some pol II genes can even increase its transcriptional rate in the presence of protein synthesis inhibitors. Besides those effects, there is also a cell response to translational arrest. Protein synthesis inhibitors have also a very potent activating effect on stress kinases, JNK, and SAPK [35] and also ERK1/2 in the presence of growth factors [36] that leads to the activation of c-fos, fos B, c-jun, junB, and jun D and transcription of genes regulated by those transcription factors. Accordingly, if the protein under study is subjected to PTM by those stress kinases, both the effect of protein synthesis inhibition and changes in PTMs of the protein, likely affecting also its PPIs, could be implicated in the rate of degradation observed under those conditions. The role of PTM and PPIs in the regulation of the degradation rate of proteins has many examples in the literature [37], and their effects on a proteomic scale are beginning to be studied [22, 29]. The point made here is that PTMs and PPIs have always to be considered as a possible mechanism that may modify the interpretation of the results obtained by the use of protein synthesis inhibitors.

Another possible situation is that the protein under study is poorly degraded even after prolonged incubation of the cells with protein synthesis inhibitors. Those results could be due to the actual fact that the protein under study has a very long half-life or alternatively that a protein with a shorter half-life is required to interact with the specific protein under study in order to be targeted to degradation. It is also noteworthy to recall that protein synthesis inhibitors also affect the machinery of degradation, with inhibitory effects on the multicomponent ubiquitin-proteasome [38–40] and autophagic pathways [41]. The combined use of protein synthesis and degradation inhibitors in time-course experiments is the most usual experimental setup to study the role of different proteolytic degradation pathways, and the considerations made above should also be taking into account for the interpretation of the results of these combined pharmacological experiments.

### 3.2. Genetic Interference Studies of Protein Degradation

Transcriptional shut-off (Tet-on, Tet-off) by tetracycline responsive promoters of protein expression [42] and mRNA translational inhibition and/or mRNA degradation using siRNA or shRNA [43] are the main genetic methods to knock-down the mRNA levels encoding for the protein whose degradation is being investigated or for determining the role of a protein in the degradation of another protein. The correct interpretation of the results of these genetic interference methods requires understanding that mRNA degradation, and/or translation inhibition, is a time-dependent process. Accordingly, a new steady state of the protein levels will be attained concomitantly with the decay of the corresponding mRNA and/or its translational inhibition, either after transcriptional shutdown of gene expression (Tet-on/off systems) or by inhibition of translation and/or degradation in the case of siRNA and shRNAs. Using the spreadsheet available on-line, the reader can model different experimental situations to simulate this type of experiments. If the half-life of the mRNA is much shorter than the actual half-life of the protein, the experimental results obtained in a shut-off transcriptional or RNA interference experiment will allow the accurate determination of the half-life of the protein. In any other situation, the mRNA half-life (and/or the rate of translational inhibition) will determine the rate of disappearance of the protein measured by immunoblot of total cell extracts. As a consequence, to correctly interpret the results obtained in both types of genetic interference setups, the rate of decay of the mRNA and/or translation inhibition (easily determined by pulse radioactive experiments and immunoprecipitation) has to be evaluated. Unfortunately, those control experiments are frequently not considered when using those methodologies. As an example, this criticism applies to the studies of alpha-synuclein half-life in cells using the Tet-on/off methodology [44, 45]; those studies do not take into account that their results on the half-life of the protein are affected by the rate of decay of the alpha-synuclein mRNA. As a consequence, their conclusions about the regulation of the degradation of alpha-synuclein are not correctly validated.

## 4. Pulse-Chase Experiments

Pulse-chase experiments are done either with the traditional radioactive aminoacids (^35^S-Met, Cys, or ^14^C aminoacids) followed by total radioactive count or immunoprecipitation with specific antibodies to the protein under study or the heavy/light isotope pulse labelling, SILAC, followed by mass spectrometry- (MS-) based quantitative proteomics (see comments above, under steady-state versus kinetic studies). Traditional pulse-chase experiments measure the rate of degradation of newly synthesized proteins. The newly synthesized proteins followed a maturation process (folding and in most cases oligomerization) and may be subjected to specific PTMs and could present unique PPIs that may differ considerably from those of the preexisting proteins (already mature proteins) in the cell. As a consequence, is conceivable that the rate of degradation of the newly-synthesized (labelled) proteins may be different from the rate of degradation of the proteins pre-existing in the cell as measured, for example, by treatment of the cells with protein synthesis inhibitors. There are factors that must be taken into account in those traditional pulse-chase experiments. Before the pulse with the radioactive aminoacids, cells are usually starved for aminoacids during variable time periods to deplete the endogenous pool of the aminoacid that will be used for protein labelling. During the starvation period cells, respond to aminoacid starvation by activating the ubiquitin-proteasomal pathway earlier than the activation of the autophagic pathway, producing an increase in protein degradation rates of pre-existing proteins in order to ensure the continuation of aminoacid supply for de novo protein synthesis [46]. Proteasome inhibitors are usually tested in this experimental setup to inhibit protein degradation by the ubiquitin-proteasome pathway, with the effects already described in the previous section. As a consequence, keeping to a minimum, the time of aminoacid starvation and pulse-radioactive labelling (total 30 min–2 h) is a good experimental practice. Furthermore, if proteasome inhibitors are used (as discussed above) it may be required to perform pulse-chase experiments in the absence and in the presence of protein synthesis inhibitors during the chase period, to control for the possible increase in mRNA abundance due to transcriptional and posttranscriptional effects of proteasomal inhibitors.

## 5. Using Tagged Proteins

**Figure 2:**
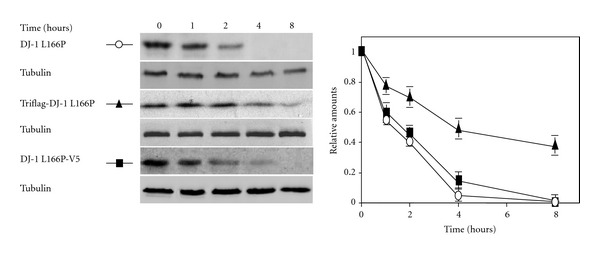
Degradation of untagged and N-terminus and C-terminus tagged versions of human DJ-1 L166P. The untagged human DJ-1 L166P (hDJ-1 L166P) construct has been described previously [49]. The C-terminal His-V5 tagged hDJ-1 L166P was obtained by PCR amplification with the following oligonucleotides (forward BamH1-DJ-1; 5′GGAAGGATCCATGGCTTCCAAAAGAGCTCTGG 3′ and reverse Nonstop-hDJ-1; 5′GTCTTTAAGAACAAGTGGCGCCTTCACTTGAGC 3′) and cloned into pcDNA 3.1/V5-His Topo vector from Invitrogen The N-terminal 3xFlag- tagged hDJ-1 L166P was obtained by PCR amplification with the following oligonucleotides (forward BamH1-hDJ-1 and reverse XhoI-hDJ-1 5′GCGCCTCGAGCTAGTCTTTAAGAACAAGTGGAGCC 3′) and cloned into the pCMV-3Tag 1-A vector. N2a cells were cultured in DMEM medium with 10% FBS and transiently transfected with the different hDJ-1 L166P constructs. Transfected cells were treated with cycloheximide (20 *μ*g/mL) for the times indicated and analyzed by Western immunoblotting with anti-hDJ-1specific antibodies, as described in [49]. Results presented are expressed as mean ± s.e.m for at least three independent experiments.

In many occasions when antibodies against the protein under study are not available, or in order to study the effect of mutations on protein stability, expression of proteins with a tag is used to study protein degradation. This technique allows easy identification of the protein by immunoblot or by immunoprecipitation with specific antitag antibodies. Another variant is the use of fusion constructs of the protein of interest with fluorescent or luminescent proteins. The general assumption behind those experiments is that the fusion of the protein of interest with any tag will show the same behaviour as the untagged protein. Any protein fused to a “big” tag, like GFP or luciferase or “medium” tags, like TAP (Tandem Affinity Purification), is “suspect” of misbehaviour from the point of view of its degradation rates, unless experimental evidence shows the opposite. This situation is especially critical when the degradation rate of the whole proteome is being evaluated with the “big” and “medium” tag methodology. The above caveat has already been pointed out in a recent publication [47], where the authors compare the estimated half-lives obtained by the ratios of GFP-fused proteins [48] and by SILAC/MS-based quantitative proteomics [19], showing big discrepancies. Another caveat with the use of tagged proteins, even if there are “small tags” as Flag, HA, Myc, V5, and so forth, is the location of the tag within the protein sequence. Most often the tag is fused either to the N- or the C-terminus of the protein under study. Very few studies take into consideration that the tag may affect the protein structure, its PTMs and PPIs or even its subcellular localization and rate of degradation. We will illustrate here the relevance of N- and C-terminal tagging with our own studies on DJ-1 degradation, a dimeric protein whose mutations are associated with early onset Parkinson's disease [49]. One of those mutations L166P disrupts dimer formation and the mutant protein has no significant secondary structure being a direct substrate for the 20S/26 proteasome [49]. In the studies of the degradation of the mutant DJ-1 L166P by cell transfection, we have compared the degradation of the untagged DJ-1 L166P with the N-terminal flag tagged and C-terminal V5-flag tag version in HeLa cells. As shown in Figure 2, the half-life of the untagged protein is rather short. Tagging the protein with Flag-tag at the N-terminus greatly increase the half-life of the protein, while tagging at its C-terminus with V5 flag produced no significant effect. Accordingly, the behaviour of N-tagged and untagged DJ-1 L166P proteins cannot be expected to be the same. In summary, if possible, for protein degradation studies (actually in any type of studies) is better to use untagged proteins and express them to levels not very different from the endogenous expression levels. When unavoidable, experiments should be performed with proteins tagged at the N- and C-terminus to clarify that the results are independent of the location of the tag.

## 6. Subcellular Localization and Protein ****Degradation

There are many examples from the literature showing that the degradation of certain proteins can take place in different cell compartments by different mechanisms, and those proteins change their subcellular localization (determined by PPIs and PTMs) after a cell stimuli or stress, and a special case, as mentioned in the introduction, is neurons [9]. The ideal experimental setup to study protein degradation will be to quantitate the rate of degradation of a protein directly within its subcellular compartment (nucleus, cytoplasm, mitochondria, etc.) by a direct method. The problem is similar to determine the actual rate of an enzyme in the cell without disrupting the cell for biochemical analysis, only NMR imaging can do it and still for a few enzymatic reactions. The use of fluorescent fusion proteins allows direct imaging and quantitation, but the caveats of this experimental approach for studying protein degradation has already being mentioned (see above), both at a proteomic scale and for studying a single protein. Nevertheless, the use of tagged proteins seems technically unavoidable at present. New semi- or noninvasive cell techniques are needed to be able to study the “in situ” degradation rates of proteins within its subcellular location in intact cells.

## 7. Checklist for Critical Appraisal of Protein Degradation Studies

A simple way to evaluate studies on protein degradation is to have in mind, or at hand, a simple scheme like the one presented in Figure 1 and to ask the following question. How many boxes of Figure 1 are not considered (lack of information) in the interpretation of the data obtained or presented? This is followed by the question: any of the processes in those boxes, black boxes in the study because of lack of information, are likely to affect interpretation of the results? The rule of the thumb is as follows: as many black boxes the study has, as much uncertainty will have the interpretation of the results. Nothing spectacular, everybody knows it.

Here we are providing a checklist for a critical appraisal of the design and interpretation of protein degradation studies. The main points to consider when analyzing a study of the role of protein degradation on the changes in protein cellular levels are as shown in Table 1.

Note that the experimental evidence obtained with the different methodologies, excluded the SILAC/MS steady-state experiments, is only partial and requires further experimental data and controls. Specific comments of the checking list for the analysis of an experiment or project on protein degradation follows.

*Tick on 1. Studying the degradation of untagged proteins is the best approach* to begin with the study of the degradation of a protein. Preferentially the endogenous levels of the protein in the cell should be studied. Alternatively, DNA constructs of the protein can be transfected in cells with a gene knock out for the protein under study. In this latter case, the expression levels of the transfected protein construct should be similar to those present in wild-type cells. The latter is also a good approach to study stability of mutant proteins in the absence of expression of the wild-type protein. The transfection experiments in knock-out cells are of little use if the lack of expression of the protein under study is known, or expected, to alter significantly the proteome or cell proteostasis by any mechanism, unless those are the objectives of the research.

*Tick on 2. Using tagged proteins is not a very good approach.* If unavoidable, perform controls with tagging at the N- and C-terminus of the protein and check if the results differ significantly.

*Ticks on 1 or 2, 3 or 4, and 7.* Tagged or untagged protein steady-state or kinetic experiments with inhibitors of protein degradation. *The changes in the protein levels cannot be attributed to changes in protein degradation rate*. Compulsory to do control experiments: to measure the amounts of mRNA, especially if proteasome inhibitors are used (they affect transcriptional rates), for the protein of interest and even the rate of translation of its mRNA. Treatment with inhibitors of protein degradation may also affect PTM of the protein under study (PPIs and subcellular localization may be also affected); remember protein kinases and phospatases are also subjected to control by degradation.

*Ticks on 1 or 2, 4, and 5.* Tagged or untagged protein kinetic experiment with inhibitors of protein synthesis. *The changes in protein levels may be attributed to protein degradation of pre-existing protein in the cell.* Additional required evidence should be provided as follows: cell viability during the time-course experiments and also that there are no changes of PTMs, PPIs, and subcellular localization of the protein under study in the presence of protein synthesis inhibitors.

*Ticks on 1 or 2, 4, 5, and 7.* Tagged or untagged protein kinetic experiment with inhibitors of protein synthesis and degradation. *The changes in protein levels may be attributed to protein degradation of pre-existing proteins in the cell.* The inhibitors of protein degradation can be added at the same time as protein synthesis inhibitors or earlier. Prolonged preincubation periods of the cells with inhibitors of degradation should be avoided, as it may change the levels of the protein due to changes in transcription, mRNA stability, translation, and so forth. Furthermore, long preincubation may also alter the expression levels of many proteins, possibly resulting in a modification of PTMs or PPIs of the protein under study, obviously affecting its rate of degradation. Always consider that PTMs, PPIs, and subcellular localization can be affected by the use of protein synthesis and degradation inhibitors.

*Ticks on 1 or 2, 4, and 8.* Tagged or untagged protein kinetic experiment with genetic interference. *The decrease in protein levels cannot be attributed exclusively to protein degradation.* Compulsory to measure the decay (time-course) of the mRNA (Tet-on/Tet-off experiments), and decay of mRNA and/or translational inhibition (RNA interference experiments), in order to be able to interpret the results. Perform other kinetic experiments to validate the data obtained. The on-line spreadsheet can be used as a help to interpret the results.

*Ticks 1 or 2, 4, 7, and 8.* Tagged or untagged protein kinetic experiment with genetic interference and inhibitors of protein degradation. *The changes in protein levels cannot be attributed exclusively to protein degradation.* Compulsory to measure rate (time-course) decay of the mRNA (Tet-on/Tet-off experiments) and decay of mRNA and/or translational rate (RNA interference experiments) in the absence and in the presence of protein degradation inhibitors. The on-line spreadsheet can be used as a help to interpret the results.

*Ticks on 1 or 2 and 6.* Tagged or untagged protein using pulse-chase radioactive experiments. *The decrease in the levels of the labelled protein can be attributed to degradation of the newly synthesised protein.* The rate of degradation of the newly synthesized protein may not be identical to the rate of the degradation of the pre-existing protein in the cell. The rates will be similar (or identical) when protein folding, PTMs, PPIs, and subcellular localization of the newly synthesized protein are much faster processes than the rate of degradation of the protein.

*Ticks on 1 or 2, 6, and 7.* Tagged or untagged protein using pulse-chase radioactive experiments with inhibitors of protein degradation. *The changes in the levels of the labelled protein can be attributed to degradation of the newly synthesised protein.* The rate of degradation of the newly synthesized protein may not be identical to the rate of the degradation of the pre-existing protein. The inhibitors of protein degradation are routinely added at the beginning of the chase period, and they can produce a change in the amount of mRNAs or mRNA translational rate (see above) producing a faster chase and decreasing the estimated value of the half-life of the protein. Addition of protein degradation inhibitors during the aminoacid starvation before the pulse or along with the pulse may be needed for proteins with short half-life (<2 h). In those cases, again, the presence of protein degradation inhibitors could affect the rate of protein synthesis and as a consequence, the initial amounts of the radioactive protein under study could be different respect to the controls. Under these experimental conditions is generally assumed that inhibitors of protein degradation do not affect protein folding, PPIs, PTM, and subcellular localization of the newly synthesized protein, but it could not be the case.

*Ticks on 1 or 2, 5, 6, and 7.* Tagged or untagged protein using pulse-chase radioactive experiments with inhibitors of protein synthesis and degradation. *The changes in the levels of the labelled protein can be attributed to degradation of the newly synthesised protein and can be compared with the degradation rate of pre-existing protein in the cell.* This experimental setup allows the comparison of the rate of degradation of the newly synthesized protein (pulse-chase) and that of the pre-existing protein by addition of the protein synthesis inhibitors during the chase period. In essence, performing immunoprecipitation experiments of radioactive total cell extracts together with determination of total protein levels by immunoblot analysis. If the degradation of the newly synthesized protein is inhibited by the presence of protein synthesis inhibitors, it could indicate that another protein(s) with a shorter half-life than the protein under study is required for targeting the protein under study to degradation. Alternatively, it could indicate that PTMs or PPIs changes in response to protein synthesis inhibitors affect the degradation of the newly synthesized protein. Apply also the same cautions respect to the use of inhibitors of protein degradation as in previous entries.

*Ticks on 1 or 2 and 9.* Tagged or untagged protein using steady-state SILAC/MS experiments. *The calculated changes in the levels of the labelled protein can be attributed to protein degradation under steady-state conditions.* By comparison of the rates of disappearance of the heavy/light and appearance of the light/heavy peptides after the shift in the SILAC experiments, the degradation rate of a protein under steady-state conditions can be estimated. This methodology seems the best suited to estimate protein turnover under unperturbed cell conditions. The half-life values obtained are average values resulting from the interplay of the rates of the different processes involved in protein homeostasis (Figure 1). At present, the results reported by different groups have strong variability that can be due to the use of different cell lines, but it might also reflect technical problems related to handling, processing of the samples, MS sequence coverage of the proteins, and also to calculations.

*Ticks on 1 or 2 and 10.* Tagged or untagged protein using kinetic SILAC/MS after cell stimulus or stress. *The calculated changes in the levels of the labelled protein cannot be attributed to protein degradation exclusively.* Any perturbation of the steady state of a cell by a stimulus or stress, like treatment of cells with inhibitors of protein synthesis or degradation, needs to show that the changes in degradation rates cannot be explained by changes in other proteostatic processes (Figure 1), changes in the protein abundance due to transcriptional, posttranscriptional processing of RNA (including mRNA transport and decay), or changes in protein synthesis, folding, oligomerization, PTMs, and PPIs as a consequence of the stimuli or stresses applied to the cells.

## 8. Conclusion

Interpretation of studies of protein degradation requires, as with other biological experiments, a critical assessment of the methodology and the data obtained. A rigorous analysis will prevent misleading conclusions. Examining the data obtained by asking a series of simple proteostatic questions can uncover serious deficiencies. Sometimes it could be difficult to interpret the data, and to present a balanced and impartial summary may be not an easy task. However, failing to do a critical analysis is damaging for science. This situation is especially relevant in a time of massive “omic” data availability. The incorrect interpretation of the enormous amounts of data obtained, together with the postdata computational analysis by merging of actual data on protein degradation with human classifications of the properties of proteins (GO terms, pI, unstructured elements, molecular weight, etc.), may produce many papers and holistic conclusions that in a few years could generate a completely new whole field of science “mislead omics.”

**Note 1**

Since this review was submitted, a report has appeared [50] describing that DJ-1 L166P promotes cell death by dissociating Bax from Bcl-XL. Unfortunately the authors used N-Terminal Flag DJ1 L166P for their experiments, and as shown here Flag-DJ-1 L166P (Figure 2) has a reduced rate of degradation compared to the untagged version, and as a consequence has higher cellular steady-state protein levels than the untagged DJ-1 L166P. As PPIs are governed by Law of Mass Action (equilibrium constant), the interactions reported for the N- Flag-DJ-1 L166P may not be quantitatively relevant for the natural (untagged) DJ-1 L166P. Accordingly, the effect of DJ-1L166P on apoptosis would be minimal or even not existent.

## Figures and Tables

**Table 1:** 

	Type of study		Conclusion
1	□	Untagged protein	Good approach
2	□	Tagged protein	Not a good approach
3	□	Usual steady state	Partial evidence
4	□	Kinetic (time-course)	Partial evidence
5	□	Protein synthesis inhibitor	Partial evidence for pre-existing proteins in the cell
6	□	Radioactive pulse-chase experiment	Partial evidence for newly synthesized proteins
7	□	Inhibitors of protein degradation	Partial evidence
8	□	Tet-on Tet-off, RNA interference	Partial evidence
9	□	SILAC-MS steady-state experiments under basal conditions (turn-over)	Good evidence
10	□	SILAC-MS upon cell stimulus or stress	Partial evidence
